# PBK/TOPK enhances aggressive phenotype in prostate cancer via β-catenin-TCF/LEF-mediated matrix metalloproteinases production and invasion

**DOI:** 10.18632/oncotarget.3709

**Published:** 2015-03-30

**Authors:** Joshua D. Brown-Clay, Deepika N. Shenoy, Olga Timofeeva, Bhaskar V. Kallakury, Asit K. Nandi, Partha P. Banerjee

**Affiliations:** ^1^ Department of Biochemistry and Molecular and Cellular Biology, Georgetown University Medical Center, Washington, DC, USA; ^2^ Departments of Oncology and Radiation Medicine, Lombardi Comprehensive Cancer Center, Georgetown University Medical Center, Washington, DC, USA; ^3^ Department of Pathology, Georgetown University Medical Center, Washington, DC, USA; ^4^ Cockeysville, Maryland

**Keywords:** PBK/TOPK, prostate cancer, metastasis, cell invasion

## Abstract

A Current challenge in prostate cancer treatment is how to differentiate aggressive disease from indolent prostate cancer. There is an urgent need to identify markers that would accurately distinguish indolent prostate cancer from aggressive disease. The aim of this study was to evaluate the role of PDZ Domain-binding kinase (PBK) in prostate cancer and to determine if PBK expression enhances aggressiveness in prostate cancer. Using archival tissue samples, gain-of-function and loss-of-function studies, we show that PBK expression is up-regulated in prostate cancer, and its expression level is commensurate with invasiveness. Modulation of PBK expression and function causally regulates the invasive ability of prostate cancer cells. Production of matrix metalloproteinases-2 and -9, which are key players in metastatic invasion, is up-regulated, and the promoters of these genes are transcriptionally activated by PBK via increased β-catenin-TCF/LEF signaling. Prostate cancer tissue specimens show that PBK's expression correlates with aggressive disease and distant metastasis in bone, lymph node and abdomen. Our i*n vitro* and *in situ* data are in agreement that PBK could be a prognostic biomarker for prostate cancer that would discriminate aggressive prostate cancer from indolent disease, and is a potential target for the therapeutic intervention of aggressive prostate cancer in men.

## INTRODUCTION

Prostate cancer is the most commonly diagnosed non-cutaneous malignancy in American men. In 2015, 220,800 men are expected to be diagnosed with prostate cancer and approximately 27,540 men are expected to die of this disease [1]. Discovery of prostate specific antigen (PSA) has revolutionized the screening process and increased the detection of prostate cancer at earlier stages; however, PSA does not effectively discriminate indolent from aggressive disease. In most cases, prostate neoplasias are found to grow quite slowly and are asymptomatic in their earlier stages. In many men, prostatic neoplasia will remain indolent and never progress to metastatic disease. Indeed, 82% of men with prostate cancer will die from causes unrelated to the cancer [2]. Accordingly, it is estimated that 1,000 men would have to be screened with the PSA test in order to prevent one death from prostate cancer, with a substantial degree of unnecessary treatment [3]. Prognostic nomograms, such as the D'Amicio risk stratification categories, which utilize a combination of stage, Gleason score from prostate biopsy and PSA level to predict the risk of recurrence and progression, do not possess sufficient accuracy to robustly predict which neoplasias are likely to progress, or continue to be indolent [4]. Therefore, there remains a strong need for the development of additional predictive markers that would be clinically useful in the treatment of prostate cancer patients [5].

The human kinome, encoding about 518 protein kinases, plays a pivotal role in normal cell physiology [6, 7]. However, the dysregulated expression or function of these kinases leads to pathological conditions and a significant fraction of them are responsible for uncontrolled growth stimulation and malignant development [8-11]. By virtue of their diverse target utilization, the kinases influence growth and differentiation by altering an expansive array of molecular switches [12, 13]. We have been focusing on a protein kinase, PDZ-binding kinase (PBK) or T-LAK cell-originated protein kinase (TOPK), which is highly homologous to the MAP kinase kinases, particularly MKK3 [14, 15]. Physiologically, PBK/TOPK plays a positive regulatory role in proper chromosomal separation and cytokinesis through phosphorylation of various targets [16, 17]. PBK/TOPK's role in facilitating proliferation is consistent with the fact that PBK/TOPK gene expression is regulated by the cell cycle-specific transcription factors, E2F and CREB/ATF [18], which undergo frequent activation in malignant tissue. PBK/TOPK is also activated post-translationally through Cdk1-cyclin B-mediated phosphorylation at Thr9 during the mitotic phase of the cell cycle [14].

While PBK/TOPK is not detectable in most human normal adult tissues, it is expressed to a significant level in certain proliferating normal cells, such as the testicular and placental tissues [14]. Analysis of PBK expression during spermatogenesis suggested that PBK is expressed at the outer cell layer of seminiferous tubules, which are populated with spermatogonia and primary spermatocytes, but not in the mature spermatozoa [19]. It is frequently up-regulated in human cancers of diverse tissue origins, although little is known about its function and even less about the functional consequences of the constitutive, higher levels seen in various cancers [20]. Ectopic PBK expression has reportedly caused attenuation of the DNA damage checkpoint at G2/M transition leading to aberrant mitosis and accumulation of polyploid progeny, mostly due to its interaction with tumor suppressor p53, destabilizing it [21]. Conversely, inhibition of PBK decreased the tumorigenic ability of colon cancer cells [22]. PBK is associated with poor prognosis of lung cancers [23]. It has recently been reported that PBK is present in primary prostate cancer [24]; however, the molecular and phenotypic implications of its presence in aggressive prostate cancer remain speculative. We wanted to examine if PBK/TOPK is differentially expressed in human prostate carcinoma, as compared to normal prostate, and the functional consequences of its expression. In the present report, we show that ectopic expression of PBK/TOPK in less aggressive prostate cancer cells greatly increased their invasive ability. Consistent with this observation is the decreasing invasiveness, following knockdown expression of PBK/TOPK, of prostate cancer cells with initially high endogenous expression. At the molecular level, we demonstrate that transcriptional activity of gene promoters of the matrix metalloproteinases (MMP) -2 and -9, which are overexpressed in aggressive carcinomas [25], is up-regulated in the presence of PBK/TOPK, as a result of PBK-dependent activation of β-catenin-TCF/LEF signaling. We corroborate our in vitro studies with a large cohort of human prostate cancer tissue samples and show a direct correlation of PBK/TOPK expression with poorly differentiated prostate cancers, and accumulation of the kinase in invasive primary tumors and particularly distant metastases. Together, we provide evidence that PBK/TOPK, by induction of a pro-metastatic gene expression program in prostate cancer cells, triggers aggressive behavior and we propose evaluation of PBK/TOPK as a prognostic marker and therapeutic target in men with aggressive prostate cancer.

## RESULTS

### PBK/TOPK expression is commensurate with the invasive properties of prostate cancer cells

**Figure 1:**
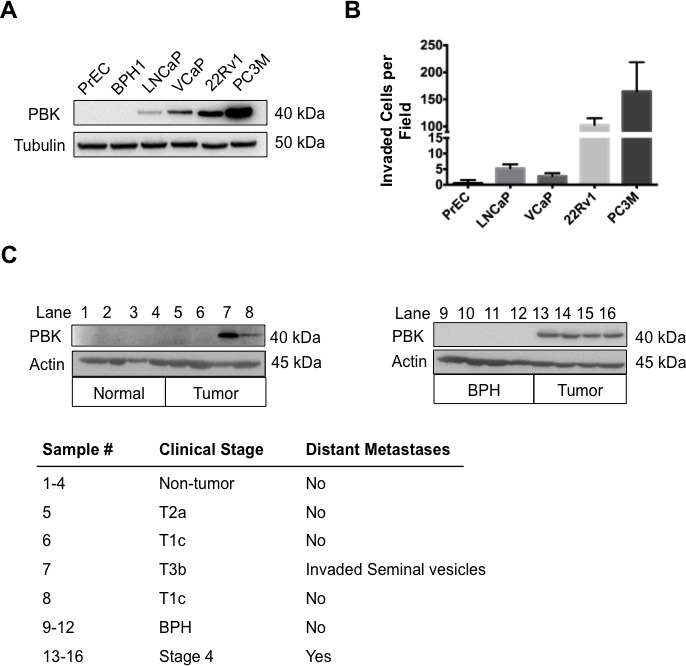
Levels of PBK in prostate cancer cell lines correlate with invasion through Matrigel (**A**) Western immunoblot assay showing PBK levels in primary normal prostate epithelial cells (PrEC), non-tumorigenic benign prostatic hyperplasia cell line (BPH-1), hormone-sensitive prostate cancer cell lines (LNCaP, VCaP), hormone-refractory prostate cancer cell line (22Rv1), and androgen receptor-negative prostate cancer cell line (PC-3M). Tubulin level was utilized as an internal control for equal protein loading. (**B**) Quantification of Matrigel invasion assay with the above cell lines showing number of cells invaded per field of view through the Matrigel matrix. The vertical axis is discontinuous for accurate presentation of high and low invasion. Four fields of view were counted and averaged for each cell line. (**C**) Western immunoblot assay showing PBK levels in human normal (non-tumorigenic adjacent to tumorigenic tissue) prostate tissue (lane 1-4), benign prostatic hyperplasia tissue (lane 9-12) and prostate cancer tissue samples (lane 5-8 and 13-16). The clinical stage for the samples is shown in the table below. *n* ≥ 3.

Given the aberrant expression of PBK/TOPK in a variety of cancer types [26], we decided to investigate the expression of PBK/TOPK in a panel of prostate cell lines. Our data demonstrate that PBK/TOPK expression correlates with aggressiveness and invasive ability in this panel of prostate cell lines (Figure 1A and 1B), including the hormone responsive LNCaP and VCaP, hormone-refractory 22Rv1 and highly aggressively metastatic derivative PC-3M [27]. BPH-1 (a SV40-immortalized hyperplastic prostate epithelial cell line) and primary prostate epithelial cells (PrEC) were included in the study as non-tumorigenic cells. PBK/TOPK expression was not detectable in PrEC or BPH-1 cells, whereas LNCaP and VCaP express low levels of PBK/TOPK, and 22Rv1 and PC-3M possess increasingly higher levels of PBK/TOPK (Figure 1A). PBK/TOPK gene expression was also analyzed via quantitative PCR and mRNA levels among the cell lines in the panel were found to be similar to the observed protein levels (Supplementary Figure 1A).

To establish if this observation was relevant to clinical prostate cancer, PBK/TOPK expression was analyzed in normal and prostate cancer samples of human origin. Normal (*n* = 4), benign prostate hyperplasia (*n* = 4) and prostate cancer (*n* = 8) samples were lysed and PBK/TOPK protein levels were determined using Western blot analyses (Figure 1C). Similar to the prostate cells (PrEC, BPH-1 and cancer cells), normal prostate and benign prostate hyperplasia displayed no detectable PBK/TOPK, while six of the eight prostate cancer samples were positive for PBK/TOPK. Interestingly, the two tissue samples that lacked PBK/TOPK were stage I and II prostate cancers while four of the six tissue samples with detectable PBK/TOPK levels were stage IV prostate cancer. PBK/TOPK expression was also analyzed via semi-quantitative RT-PCR and this data closely matched PBK/TOPK protein levels (Supplementary Figure 1B), thereby confirming that PBK/TOPK is expressed exclusively in tumor samples and not in normal prostatic tissue. These data show that PBK expression correlates well with the clinical phenotype, in addition to its correlation with *in vitro* invasiveness observed in prostate cancer cell lines.

### Invasive properties of prostate cancer cells are modulated by ectopic expression of PBK or knockdown of PBK expression

**Figure 2:**
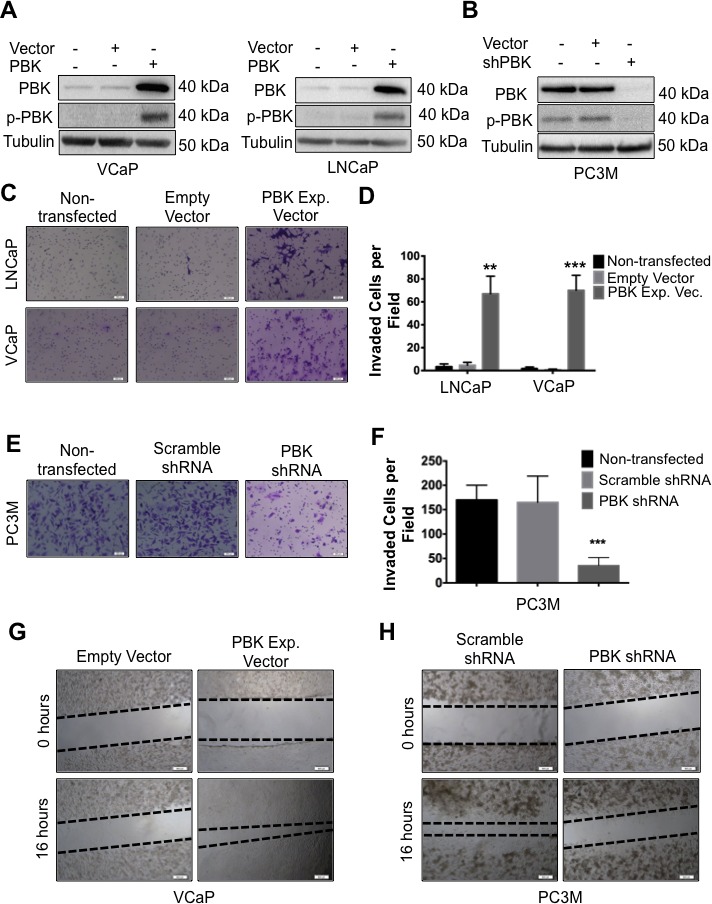
PBK causally modulates invasive and migratory potential of prostate cancer cells (**A**) Ectopic expression of PBK in hormone-sensitive LNCaP and VCaP cells is measured by Western blot analysis. Non-transfected and empty vector-infected cells were used as controls. (**B**) Western blot showing knockdown of PBK in PC-3M cells. Non-transfected and scrambled shRNA-transfected cells were used as controls. Representative images of (**C**) LNCaP or VCaP cells, either appropriate controls or stably overexpressing PBK, stained with crystal violet after being subjected to a modified Boyden chamber invasion assay, in addition to (**E**) PC-3M cells, with PBK expression stably knocked down and appropriate controls. (**D** and **F**) Quantification of cells that had invaded from three different experiments. Invaded cells were counted in four fields of view from each experiment. Quantitative data are represented as SEM ± SE. ** represents a *p*-value <0.01 and *** a *p*-value <0.001. Representative images of cell migration 16h after creation of a wound in (**G**) VCaP-PBK and (H) PC-3M-shPBK stably modified cells compared to their respective controls (empty vector or scrambled-shRNA stable cells, respectively). The black dotted line indicates the boundary of the area covered by cells. *n* ≥ 3.

The observation that PBK/TOPK expression level is commensurate with the invasive properties of prostate cancer cells prompted us to examine if prostate cancer cells with low endogenous PBK/TOPK show increased invasiveness upon ectopic expression of PBK/TOPK. To this end, VCaP and LNCaP cells, which express low levels of PBK/TOPK, were infected with a PBK/TOPK expression vector and stable cell lines overexpressing PBK/TOPK were isolated (Figure 2A). We demonstrate that PBK/TOPK overexpression resulted in an increased invasiveness of the transfected clones, compared to parental cells and empty vector-infected controls (Figures 2C and 2D). Interestingly, upon overexpression of PBK/TOPK, VCaP cells acquired a spindle-shaped morphology, a characteristic of more aggressive cell type (Supplementary Figure 2A).

Conversely, PC-3M cells, which express significantly higher levels of PBK/TOPK and are highly invasive compared to VCaP and LNCaP cells, were transfected with a silencing PBK/TOPK shRNA vector, causing abrogation of PBK/TOPK expression (Figure 2B). In contrast to VCaP cells overexpressing PBK/TOPK, silencing of PBK/TOPK in PC-3M cells changed their previous spindle-like epithelial morphology to a more flattened one with increased cytoplasm (Supplementary Figure 2B). These cells had significantly decreased invasive ability, compared to parental cells or scrambled shRNA-transfected cells (Figures 2E, 2F).

Figures 2G and 2H show the results of an *in vitro* wound-healing assay. Narrowing gaps between dotted lines demonstrate cell migration, which is increased upon ectopic PBK/TOPK expression in VCaP cells while knockdown of PBK/TOPK expression in PC-3M cells decreased cell migration. Together, these data indicate that PBK/TOPK causally regulates the migratory and invasive ability of prostate cancer cells.

To determine if PBK/TOPK protein levels had any effect on cell proliferation, we examined cell growth in the presence or absence of PBK/TOPK (Supplementary Figure 3). Overexpression of PBK/TOPK in VCaP cells increased cell proliferation significantly, while the loss of PBK/TOPK in PC3M cells reduced cell proliferation more strongly, suggesting that PBK/TOPK levels are also associated with the proliferative potential of prostate cancer cells.

### Metalloproteinases MMP-2 and MMP-9 levels are modulated by PBK/TOPK

**Figure 3:**
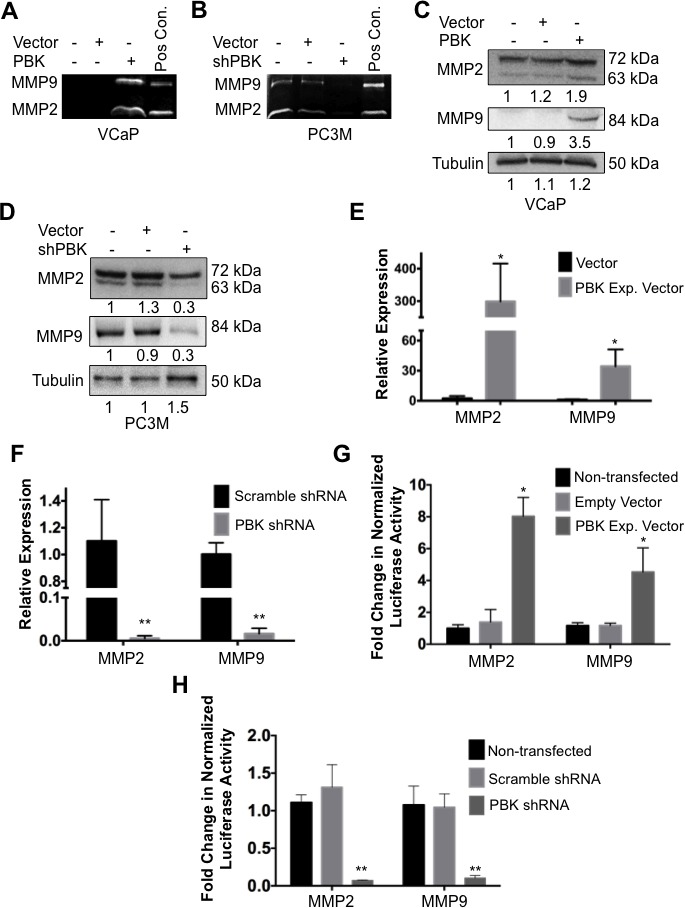
PBK modulates expression of metalloproteinases MMP-2 and MMP-9 Gelatin zymographies of cell-conditioned media from (**A**) VCaP cells stably overexpressing PBK and (**B**) PC-3M cells with PBK stably knocked down, with appropriate controls. Conditioned media from HT1080 fibrosarcoma cells was used as a positive control (Pos Con) for MMP-2 and −9. Western immunoblots for MMP-2 and MMP-9 in cell extracts from (**C**) VCaP and (**D**) PC-3M. qPCR analyses comparing MMP-2 and MMP-9 mRNA levels in (**E**) PBK-overexpressing VCaP and (**F**) PBK knockdown PC-3M cells, along with their respective controls. Luciferase activities of MMP-2 and MMP-9 promoters in (**G**) PBK-overexpressing VCaP and (H) PBK-knocked down PC-3M cells were compared with appropriate controls. * represents a *p*-value < 0.05, and ** a *p*-value < 0.01. *n* ≥ 3.

Matrix metalloproteinases MMP-2 and MMP-9 are implicated in tissue invasion in metastatic malignancies [28]. Consequently, we examined the effect of PBK/TOPK expression on MMP-2 and -9 levels by measuring gelatinolytic activities in cell-conditioned media and by immunoblot assays. Figures 3A-3D show that PBK/TOPK expression upregulates MMP-2 and -9 gelatinolytic activity of conditioned media and their protein levels as well.

To determine if alterations in MMP protein levels are due to similar changes coordinated by PBK/TOPK at the transcript level, quantitative PCR was performed. Figures 3E and 3F show that the accumulation of MMP-2 and -9 mRNAs is significantly increased in the presence of PBK/TOPK expression, as indicated by quantitative PCR analyses, whereas, MMP-2 and -9 mRNAs are significantly decreased after PBK/TOPK knockdown as compared to appropriate controls. To determine if this is due to increased transcriptional activation at the promoters of these genes, VCaP and PC-3M cells were transfected with MMP-2 and -9 promoter luciferase reporter constructs. We observed that transcription from the MMP-2 and -9 promoters is positively regulated by PBK/TOPK expression (Figure 3G). Conversely, transcription from the MMP-2 and -9 promoters is decreased by knockdown of PBK/TOPK (Figure 3H). Taken together, these data show that PBK/TOPK modulates MMP-2 and -9 levels in conditioned media through transcriptional upregulation of the genes.

### PBK/TOPK enhances cell invasiveness via β-catenin-TCF/LEF signaling

**Figure 4:**
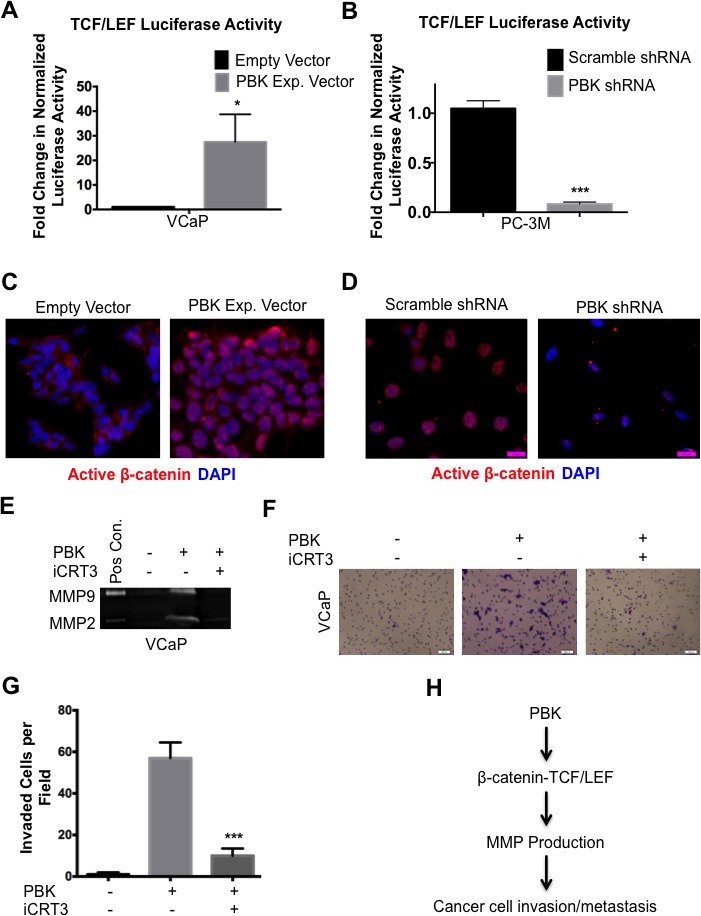
β-catenin/TCF/LEF signaling mediates PBK-dependent invasion in prostate cancer cells TCF/LEF-luciferase reporter activities in (**A**) VCaP cells stably overexpressing PBK and (**B**) PC-3M with stably knocked down PBK and PC-3M cells, with appropriate controls. Immunofluorescence of stably modified (**C**) VCaP and (**D**) PC-3M cells showing localization of active (non-phosphorylated) β-catenin. Scale bars are 100 μm. Gelatin zymography showing reduction of PBK-induced MMP-2 and MMP-9 gelatinolytic activities (**E**) and invasion (**F**) in VCaP cells after pharmacological inhibition of β-catenin/TCF/LEF signaling. PBK-overexpressing VCaP cells were treated with iCRT3 (an inhibitor of β-catenin-/TCF/LEF interaction; 50 μM) for 24 h. Conditioned media from HT1080 fibrosarcoma cells was used as a positive control (Pos Con) in the gelatin zymography. (**G**) Quantification of cells that invaded through Matrigel was counted from four representative view fields of three independent experiments. * represents a *p*-value < 0.05, ** a *p*-value < 0.01, and *** a *p*-value < 0.001. (**H**) Schematic showing our hypothesis that PBK enhances the invasive ability of prostate cancer cells through activation of β-catenin/TCF/LEF signaling and subsequent upregulation of MMP-2 and -9.

Since PBK/TOPK is a protein kinase and has not been reported to have any ability to directly regulate gene expression, it seemed likely that it was effecting its transcriptional changes through regulation of a signaling pathway. To this end, we investigated the activation status of multiple signaling pathways (data not shown). The pathway that we found most consistently and strongly activated in the presence of PBK/TOPK in multiple prostate cancer cell lines was that of β-catenin-TCF/LEF signaling. VCaP, PC-3M and their stably transfected derivatives were transiently transfected with TCF/LEF reporter constructs. Overexpression of PBK/TOPK in VCaP cells increased TCF/LEF transcriptional activity (Figure 4A) and loss of PBK in PC-3M cells decreased TCF/LEF transcriptional activity (Figure 4B). These results were corroborated using another TCF/LEF reporter construct, TOP-FLASH (Supplementary Figures 4A and 4B).

To confirm that canonical β-catenin signaling was indeed active, we examined levels of active (non-phosphorylated) β-catenin in control and stably modified VCaP and PC3M cells. Indeed, overexpression of PBK/TOPK in VCaP cells resulted in a robust nuclear accumulation of active β-catenin (Figure 4C, also see Supplementary Figure 4C), while loss of PBK/TOPK in PC-3M cells caused a loss of both nuclear and total active β-catenin, as expected (Figure 4D, Supplementary Figure 4D).

To demonstrate that PBK/TOPK-dependent invasion is mediated through β-catenin signaling, we performed rescue experiments, wherein VCaP cells stably overexpressing PBK/TOPK were treated with a pharmacological inhibitor (iCRT3), which inhibits the interaction between β-catenin and its transcriptional coactivators, TCF/LEF, and then subjected the cells' conditioned media to gelatin zymography or the cells to a Matrigel invasion assay (Figures 4E-4G). Dosage of this inhibitor (iCRT3) [29] was determined by measuring its effect on TOP-FLASH luciferase activity, and a downstream target gene, cyclin D1, by Western blot analysis (Supplementary Figures 4E and 4F). Notably, inhibition of β-catenin signaling reduced gelatinolytic activity of conditioned media and invasive ability of PBK-overexpressing VCaP cells to almost control levels, showing that β-catenin signaling mediates the upregulation of PBK/TOPK-dependent invasion in prostate cancer cells. A schematic of this model is shown in Figure 4H.

### Pharmacological inhibition of PBK/TOPK mimics knockdown expression and modulates downstream signaling

**Figure 5:**
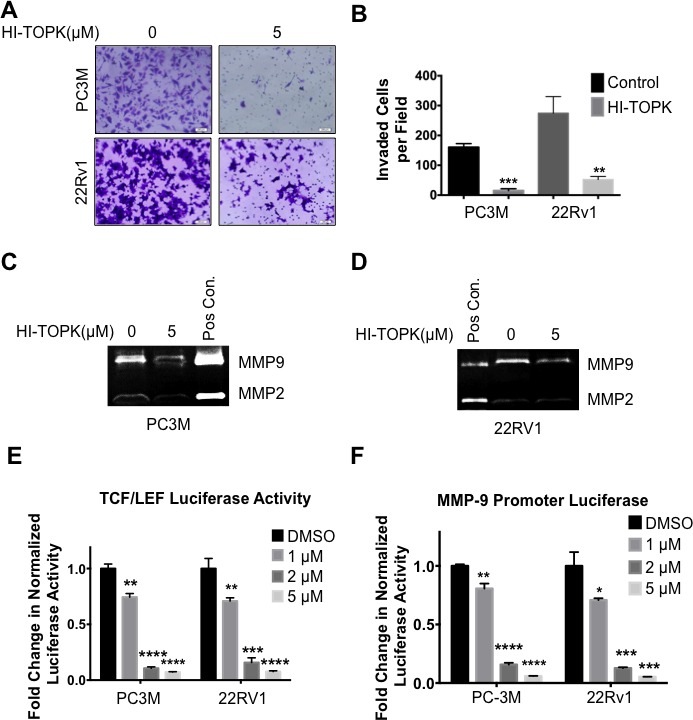
Pharmacological inhibition of PBK mimics the knockdown effect seen in prostate cancer cells (**A**) PC-3M or 22RV1 cells were treated with 5 μM of HI-TOPK-032 or DMSO (as control) for 12 hours and then subjected to a modified Boyden chamber invasion assay for the period of time as described in Materials and Methods and subsequently stained with crystal violet. (**B**) Quantification of invaded cells from four representative fields of view from three independent experiments. Gelatin zymographies using conditioned media of HI-TOPK-032-treated or DMSO-treated (control) (**C**) PC-3M or (**D**) 22Rv1 cells, respectively. (**E**) Similar to genetic knockdown, pharmacological inhibition of PBK decreases β-catenin-TCF/LEF luciferase activity, in both PC-3M and 22Rv1 cells, as well as (**F**) MMP-9 promoter luciferase activity. Cells were transfected with TCF/LEF or MMP-9 promoter luciferase reporter construct, respectively, for 48 hours and then treated with 5 μM for 24 hours, prior to being lysed and luciferase activity measured. * represents a *p*-value < 0.05, ** a *p*-value < 0.01, *** a *p*-value < 0.001, and **** a *p*-value <0.0001. *n* ≥ 3.

An ATP-competitive inhibitor of PBK/TOPK, HI-TOPK-032, has been reported to reduce the growth of colon cancer cells [30]. Consequently, we used HI-TOPK-032 to determine if the downstream effects of PBK/TOPK knockdown expression could be replicated by treatment with this inhibitor. To this end, PC-3M and 22Rv1 prostate cancer cells, both of which express high levels of PBK and have high invasive ability, were treated with the inhibitor. Interestingly, the PBK/TOPK inhibitor was found to be efficacious in causing more than a five-fold inhibition of invasion in both PC-3M and 22Rv1 cells (Figures 5A and 5B). Use of 22Rv1 cells along with PC-3M cells demonstrates that in both cell lines inhibition of PBK/TOPK decreases invasive potential and this effect is not cell line specific. Similarly, MMP-2 and -9 levels, as measured in the conditioned media, were significantly reduced in the presence of HI-TOPK-032, in both prostate cancer cell lines (Figures 5C and 5D). Conditioned media from a fibrosarcoma cell line, HT1080, was used as a positive control for MMP-2 and -9. TCF/LEF luciferase reporter activity was also reduced dose-dependently in both PC3M and 22Rv1 cells after HI-TOPK-032 treatment (Figure 5E). Similar results were also observed when MMP-9 promoter activity was monitored in the presence of PBK/TOPK inhibitor (Figure 5F). It is also interesting to note that PBK/TOPK inhibitor was likewise able to inhibit gelatinase activity in a fibrosarcoma cell line, HT1080, which expresses very high levels of MMP-2 and -9 (Supplementary Figure 5).

The effect of the pharmacological inhibitor of PBK/TOPK on cell growth was also examined. Non-toxic concentrations of HI-TOPK-032 inhibited growth of VCaP, 22Rv1 and PC-3M, as determined by MTT assay (Supplementary Figure 6). Together, these data suggest that, similar to genetic knockdown, pharmacological inhibition of PBK strongly reduces two hallmarks of cancer in prostate cancer cells, namely, invasive ability, via down-regulation of β-catenin-mediated production of MMP-2 and -9, and proliferation.

### Immunohistochemical analyses of prostate cancer tissue microarrays show abundance of PBK/TOPK in high-grade carcinoma and distant metastasis

**Figure 6:**
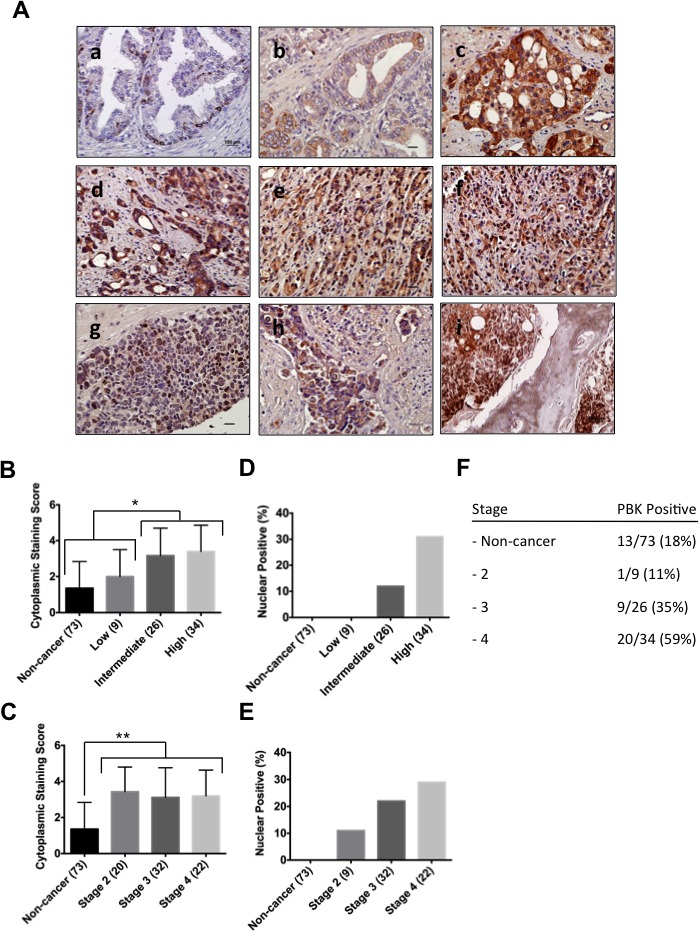

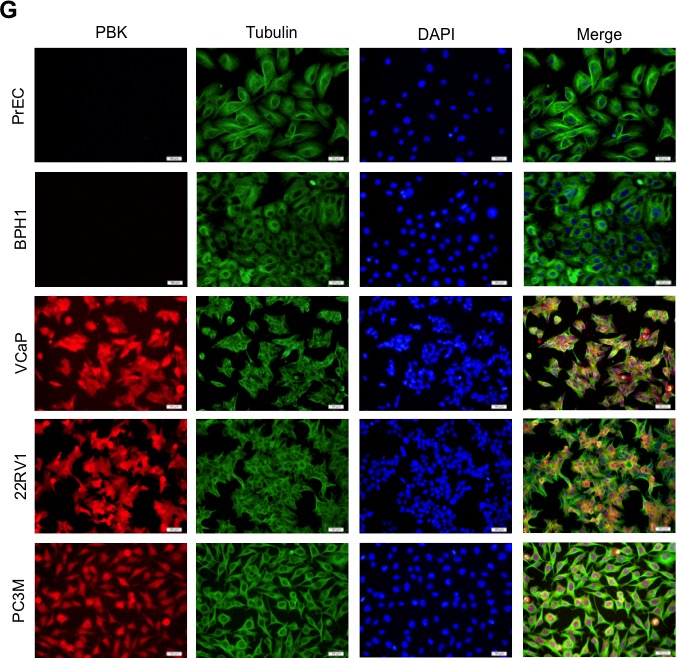
PBK protein levels and localization in human prostate cancer tissue samples and in distant metastasis (**A**) Human prostate cancer samples were immunostained with PBK-specific antibody and counterstained with hematoxylin: (a) Normal prostate tissue (non-tumorigenic adjacent to tumor tissue), (b) low-, (c) intermediate- and (d-f) high grade prostate cancer, as well as prostate cancer metastases to the (g) abdominal wall, (h) lymph node and (i) bone. Scale bars are 100 μm. The correlation between the (**B**) cytoplasmic staining score and Gleason score (*n* = 120), as well as the (**C**) correlation between cytoplasmic staining score and prostate cancer stage was determined. (**D** and **E**) represent quantification of percentage of PBK-positive nuclei in various grades and stages, respectively. (**F**) Showing the total percentages of PBK positivity among tumor samples in the tissue array. (**G**) Immunofluorescence staining of PBK in normal primary prostate epithelial cells (PrEC), BPH-1 (non-tumorigenic cell line), and prostate cancer cell lines (VCaP, 22Rv1 and PC-3M). Note nuclear localization of PBK in prostate cancer cells along with some cytoplasmic staining. * represents a *p*-value < 0.05, and ** a *p*-value < 0.01.

The clinical relevance of our study of the role of PBK/TOPK expression in prostate cancer was examined by analyzing a large patient cohort (*n* = 120). Tumor-adjacent normal tissues and prostate cancer sections with low Gleason score were frequently negative or possessed weak PBK/TOPK protein expression (Figure 6A: a, b). Tissue sections from tumors with higher Gleason score and, hence, more aggressive disease had much stronger PBK/TOPK levels (Figure 6A, c-i). Higher PBK/TOPK level was significantly associated with intermediate and high Gleason scores, as well as being significantly higher in any stage cancer sample, compared to tumor adjacent non-tumor tissue (Figure 6, B-C). We also observed that nuclear PBK localization strongly correlated with Gleason score and cancer stage (Figure 6, D-E). Most interestingly, metastatic lesions of prostate cancer in abdominal wall, lymph node and bone (Figure 6A: g-i) show robust PBK/TOPK levels and nuclear localization. We were also intrigued to see some positive immunostaining for PBK/TOPK in the basal layer of epithelial cells in non-tumor tissue sections adjacent to cancer tissue, suggesting a possible role for PBK/TOPK in this cell type (Figure 6A, a).

To confirm the PBK/TOPK localization further, prostate cancer cells lines, along with non-tumorigenic BPH-1 and PrEC, were analyzed (Figure 6G). In concordance with this finding in human prostate cancer tissue samples, non-tumorigenic primary prostate epithelial (PrEC) and BPH-1 cells had no detectable PBK/TOPK protein expression, whereas, in the tumorigenic cell lines VCaP, 22Rv1 and PC-3M, PBK/TOPK protein was localized predominantly in the nucleus, but also displayed some cytoplasmic staining (Figure 6G and Supplementary Figure 7), similar to what we observed in the human prostate cancer tissue samples.

Together, our data provide strong evidence that PBK/TOPK is present in prostate cancer cells and not in normal non-tumorigenic cells and its level increases with distant metastasis and invasive ability.

## DISCUSSION

Determining which prostate neoplasia will progress to metastatic disease is a particularly important clinical and research topic. While the five-year patient survival rate for localized and regional prostate cancer is nearly 100%, that for patients with metastatic prostate cancer is only 28% [31]. Metastatic progression simultaneously complicates patient treatment, necessitating the use of systemic therapies, while being associated with a poor prognosis. Although there has been considerable debate lately on reducing screening to minimize overtreatment of indolent prostate cancer, early detection of tumors is vital to allow treatment to begin when it would be most efficacious. Therefore, being able to distinguish aggressive prostate neoplasms from indolent ones would greatly reduce overtreatment, while maintaining the ability of clinicians to focus on aggressive, early-stage tumors. The clinical implications of grade, stage and PSA level have been substantially analyzed but are not robust enough to serve well for specifying a treatment strategy. Therefore, additional prognostic factors are desirable and molecular markers are particularly underrepresented in the diagnosis and treatment of prostate cancer.

To determine if PBK/TOPK is associated with invasive prostate cancers, we began by examining PBK/TOPK expression in a panel of prostate cancer cell lines. We observed that total PBK/TOPK protein expression correlates well with the invasive ability of the prostate cancer cell lines. We found that a very high percentage (59%) of prostate cancers with distant metastases express high levels of PBK/TOPK and ~30% of these cancers have predominantly nuclear localization of PBK/TOPK. Our results are in good agreement with two earlier studies of lung cancer where either 59% [32] or 54% [23] of stage III-IV lung cancer samples were shown to have PBK/TOPK expression. Our results also corroborate with the finding of Shiraishi et al. [24] that PBK/TOPK expression was significantly associated with prostate cancer. The most significant outcome of our studies is a very strong correlation of PBK/TOPK nuclear localization with cancer grade and stage and particularly its association with distant metastases at various sites. Such correlation suggests that total PBK/TOPK expression, and its localization, may have important clinical implications. However, future studies would be required to determine the biological significance of the nuclear versus cytoplasmic localization of PBK/TOPK.

We were fascinated to see the presence of PBK/TOPK in basal epithelial cells in non-tumor tissue sections. Although there are conflicting reports [33, 34], basal cells in the prostatic acini contain a stem cell population that is able to recreate both prostate epithelial cell layers [35]. It is interesting to note that PBK/TOPK is present in spermatogonial germ cells [19] and placenta [14], and absent in adult epithelial cell types [15], suggesting a possible role of PBK/TOPK in maintenance of a stem/progenitor cell type.

To find a molecular explanation for the increased invasion as a function of PBK/TOPK expression, we examined vital downstream effectors, such as the matrix metalloproteinases, MMP-2 and -9, which together are known to play a key role in metastatic development in human cancer patients [25]. Our data show up-regulated expression of MMP-2 and -9 in the presence of over-expressed PBK/TOPK and that expression from MMP-2 and -9 gene promoters are modulated by PBK/TOPK, indicating that transcriptional control mechanisms for these genes are positively regulated by PBK/TOPK. These matrix metalloproteinases have been demonstrated to be involved in the penetration and invasion through the basement membrane and the endothelial barriers in vivo, allowing invading cells to appear in circulation. As such, we demonstrated that PBK/TOPK levels causally regulate invasion in human prostate cancer cells in vitro. Additionally, we observed strong PBK/TOPK localization in human prostate cancer bone, lymph node and abdominal metastases. Therefore, it may be reasonable to think that PBK/TOPK is likely to contribute to metastasis in human prostate cancer to distant organs. However, it remains to be seen in an in vivo mouse model if prostate-specific expression in PBK/TOPK drives the development or progression of metastatic prostate cancer.

Given that androgen receptor (AR) signaling is a key driver of the tumor phenotype in both treatment-naïve [36] and castration-resistant prostate cancer [37, 38], we investigated if AR is able to regulate PBK expression. There is no consensus AR-response element in the promoter region of PBK, and treatment of VCaP and 22Rv1 cells with dihydrotestosterone and/or a potent inhibitor of AR signaling, enzalutamide, had no effect on PBK expression (data not shown). Therefore, although PBK expression is strongly associated with higher-grade prostate cancer, we hypothesize that PBK's expression is driven by a factor other than the AR.

Our results indicate that activation of β-catenin-TCF/LEF signaling is critical for PBK/TOPK-dependent prostate cancer cell invasion. The role of β-catenin in prostate cancer has not been conclusively confirmed. It has been shown that β-catenin plays a key role in facilitating invasiveness and metastasis in prostate cancer cell lines both in vitro [39] and in vivo [40]. In the patient cohort we analyzed, PBK/TOPK is associated with poorly differentiated prostate cancer, its nuclear localization is strongly correlated with metastatic prostate cancer, and β-catenin is important for mediating the aggressive outcome of PBK expression in vitro. Altogether, our results suggest that PBK-β-catenin signaling is important, at least in a subset of human prostate cancers.

Treatment with HI-TOPK-032 mimicked our observations in genetic knockdown cells, showing that PBK/TOPK is amenable to pharmacological inhibition. Indeed, recent work by Matsuo and associates show that a novel inhibitor of PBK/TOPK was sufficient to induce complete regression of tumors in a lung cancer xenograft model [41]. Taken together, these results suggest that targeting PBK/TOPK by pharmacological agent is sufficient to ameliorate multiple facets of the tumor phenotype, and therefore, PBK/TOPK is a potentially important therapeutic target.

In summary, the in situ analyses with prostate cancer tissue microarrays, combined with molecular measurements of resected tissue, support the observation that PBK/TOPK expression correlates with aggressive disease. Our in vitro data have been consistent with a role for PBK/TOPK in facilitating invasion in prostate cancer. Thus, we have been able to experimentally establish, for the first time, a direct role and a molecular mechanism for PBK/TOPK to facilitate aggressiveness in prostate cancer cells. Future studies in this direction are needed to shed more light on the significance of nuclear localization of PBK/TOPK, for example, the mechanism of action leading to up-regulation of PBK/TOPK-responsive gene targets that contribute to the development of aggressive, metastatic disease. The up-regulated expression of PBK/TOPK in metastatic human prostate cancer samples, in addition to the role it plays in facilitating invasive ability and cell growth of prostate cancer cell lines, raises the possibility of utilizing PBK/TOPK both as a discriminatory biomarker correlated with aggressive prostate cancer and as a key therapeutic target.

## MATERIALS AND METHODS

### Cell lines, plasmids and reagents

LNCaP, VCaP, 22Rv1, PC-3M and HT1080 (American Type Culture Collection, Manassas, VA) and BPH-1 (cells were obtained from Dr. Simon Hayward, Vanderbilt University) cells were cultured in phenol red–free Improved Minimum Essential Medium (IMEM; Life Technologies, Grand Island, NY) containing 10% FBS (Atlanta Biologicals, Flowery Branch, GA), 2 mmol/L glutamine, 100 U/mL penicillin G sodium, and 100 μg/mL streptomycin sulfate (Sigma-Aldrich, St. Louis, MO), at 37°C and 5% CO_2_. Primary prostate epithelial cells (PrEC) were obtained from Lonza (Walkersville, MD) and were cultured in K-SFM with L-Glutamine, EGF and BPE (Invitrogen). Two different PBK shRNA, and one scramble shRNA, constructs (Origene, Rockville, MD) were transfected into PC-3M cells to create stable PBK knockdown and scramble vector transfected control cells, respectively. To overexpress PBK, pLenti-suCMV-RFP-2A-Puro (GenTarget Inc., San Diego, CA) was infected into VCaP and LNCaP cells to create stable cell lines and pLenti-suCMV-null-RFP-puro (GenTarget) was used to create control cells. Forty-eight hours after transfection or infection, cells were selected with puromycin, individually cloned, and screened for loss or gain of PBK expression. All cell lines used were tested and authenticated at the Tissue Culture Shared Resource in Lombardi Comprehensive Cancer Center by DNA fingerprinting short-tandem repeat (STR) analysis.

HI-TOPK-032 was obtained from Tocris Bioscience (Bristol, UK).

### Western blot analysis

Western blots were performed as previously described [42]. Protein derived from samples of human prostate tissue was obtained from Georgetown University Tissue Core with approved IRB protocol. Thirty micrograms of protein were loaded and membranes were probed with antibodies against PBK (#2286-1 Epitomics, Burlingame, CA), MMP9 (#3852, Cell Signaling, Danvers, MA), actin, MMP2 (#47772, 10736, respectively; Santa Cruz Biotechnology, Dallas, TX), and tubulin (Sigma Aldrich #T5168).

### MMP-2 and -9 promoter activity assays

Luciferase assays were performed as previously described [42]. VCaP or PC-3M cells were transiently transfected with MMP-2 and MMP-9 promoter luciferase constructs [43], TCF/LEF-Luciferase (Promega Corp., Madison WI) or pGL4 empty vector (Promega) constructs, as well as a control *Renilla* luciferase reporter plasmid (Promega). Forty-eight hours after transfection, luciferase activity was measured in cell lysates using the Dual Luciferase Assay kit (Promega), following the manufacturer's protocol, and normalized to *Renilla* luciferase activity.

### Gelatin zymography

The gelatin zymography protocol has been described elsewhere [44]. Equal numbers of cells were plated in 10 cm plates. Once the cells had attached, the media was replaced with half the normal volume of serum-free media. The cells were incubated with the media for 16-18 hours and the media was collected. LNCaP- and VCaP-conditioned media required concentration using Amicon Ultra-0.5 Centrifugal Filter Unit with Ultracel-10 membrane (#UFC5010BK, Millipore, Billerica, MA) and 10 μl of concentrated, conditioned media were loaded into the gelatin zymography. For PC3M-conditioned media, 25 μl of non-concentrated media were loaded directly into the gelatin zymography. For HT1080-conditioned media, 10 μl of non-concentrated media were loaded directly into the gelatin zymography.

### Reverse transcriptase-PCR

RNA was extracted from LNCaP, VCaP or PC-3M cells using Trizol (Invitrogen) and converted to cDNA and measured via quantitative PCR, as described previously [42]. RNA derived from samples of human prostate tissue was obtained from Georgetown University Tissue Core with approved IRB protocol. Semi-quantitative, one-step reverse transcriptase PCR was performed using the Verso One-Step RT-PCR kit (Thermo Scientific, Waltham, MA), according to the manufacturer's instructions. Primers and conditions are described in supplemental methods.

### Immunofluorescent staining

Immunofluorescence staining was performed as described previously [42]. Active β-catenin antibody was purchased from Millipore (cat. # 05-665).

### Immunohistochemistry

Immunohistochemistry (IHC) was carried out on prostate cancer tissue microarrays purchased from US Biomax (cat. # PR956b and 8011, US Biomax, Rockville, MD) as previously described [45] using an anti-PBK Cell Signaling antibody (#4942, 1:100). The stained slides were then analyzed with a pathologist, a Gleason score determined for the available portion of the biopsy and a cytoplasmic staining score was obtained by summing two separate scores for distribution (negative = 0; focal or < 10% positive = 1; regional or 11-50% positive = 2; diffuse or > 50% positive = 3) and intensity (negative = 0; weak = 1; moderate = 2; intense = 3). A median value for PBK expression was found (3) was found and any samples possessing at least that degree of PBK expression were considered positive for PBK expression. Additionally, if any nuclear PBK was observed, that sample was considered nuclear positive.

### Cell invasion and migration assays

For LNCaP, VCaP and 22Rv1 cells, a 0.1 ml suspension of 20,000 cells (for PC-3M cells, only 10,000 cells were used) was subjected to the Boyden chamber (BD Biosciences, San Jose, CA), as described previously [46]). Cells were treated with 5 μM of HI-TOPK-032 for 12 hours prior to addition of cells to the modified Boyden chamber and HI-TOPK-032 was present for the duration of the assay as well. PC-3M cells were allowed to invade for 20 hours before fixation, while 22Rv1 and VCaP cells were allowed to invade for 24 and 72 hours, respectively. All cells were treated with mitomycin (10 ng/ml) before and during plating into Boyden chamber to inhibit cell proliferation. After crystal violet staining, pictures were taken of four representative fields of view and invaded cells were counted.

Stably genetically modified cells were grown in 24-well plates. Once cells had reached 90% confluency, a scratch was made in a representative region using a sterile 200μl pipette tip. The plates were washed two times with sterile PBS and 500 μl of complete media further added to the plate. The plates were left for 16 hours and photographs were taken under inverted microscope.

### Statistical analysis

All data were derived from at least three independent experiments and statistical analysis was conducted using the Prism 3 GraphPad software. Values were presented as means ± SEM. Significance level was calculated using Student's t-test or ANOVA, as appropriate, for the in vitro experiments, while the Kruskal-Wallis test was used to determine if the groups in the immunohistochemistry experiments were significantly different. A p value <0.05 was considered significant. Once significance was established for the results of the immunohistochemistry experiments, the Mann-Whitney test was used to establish which groups differed significantly from the others.

## SUPPLEMENTARY MATERIAL AND FIGURES


