# Erratum: PBK/TOPK enhances aggressive phenotype in prostate cancer via β-catenin-TCF/LEF-mediated matrix metalloproteinases production and invasion

**DOI:** 10.18632/oncotarget.6865

**Published:** 2016-01-09

**Authors:** Joshua D. Brown-Clay, Deepika N. Shenoy, Olga Timofeeva, Bhaskar V. Kallakury, Asit K. Nandi, Partha P. Banerjee

***Oncotarget. 2015; 6:*** *15594-15609*

***doi:*** *10.18632/oncotarget.3709*

**Present Figure 2:** During the assembly of figure 2C, the same image was inadvertently used for both the non-transfected and empty vector control.

**Figure 2:**
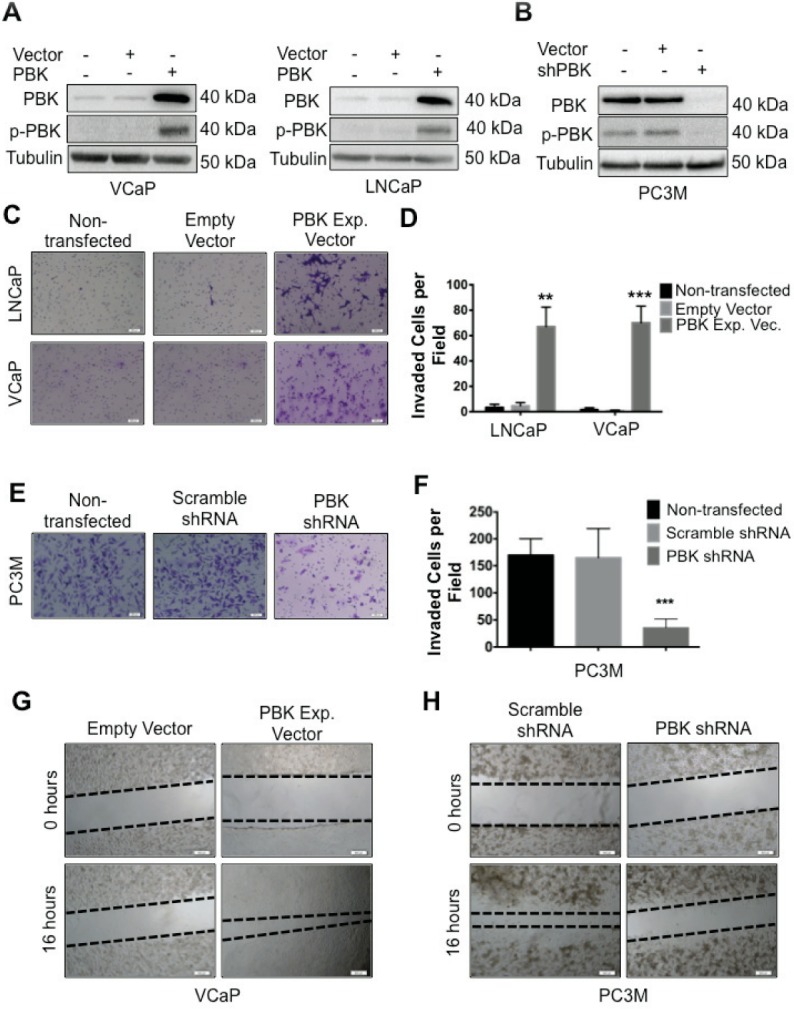
PBK causally modulates invasive and migratory potential of prostate cancer cells (**A**) Ectopic expression of PBK in hormone-sensitive LNCaP and VCaP cells is measured by Western blot analysis. Non-transfected and empty vector-infected cells were used as controls. (**B**) Western blot showing knockdown of PBK in PC-3M cells. Non-transfected and scrambled shRNA-transfected cells were used as controls. Representative images of (**C**) LNCaP or VCaP cells, either appropriate controls or stably overexpressing PBK, stained with crystal violet after being subjected to a modified Boyden chamber invasion assay, in addition to (**E**) PC-3M cells, with PBK expression stably knocked down and appropriate controls. (**D** and **F**) Quantification of cells that had invaded from three different experiments. Invaded cells were counted in four fields of view from each experiment. Quantitative data are represented as SEM ± SE. ** represents a *p*-value <0.01 and *** a *p*-value <0.001. Representative images of cell migration 16h after creation of a wound in (**G**) VCaPPBK and (H) PC-3M-shPBK stably modified cells compared to their respective controls (empty vector or scrambled-shRNA stable cells, respectively). The black dotted line indicates the boundary of the area covered by cells. *n* ≥ 3.

**Corrected Figure 2**:

**Figure 2:**
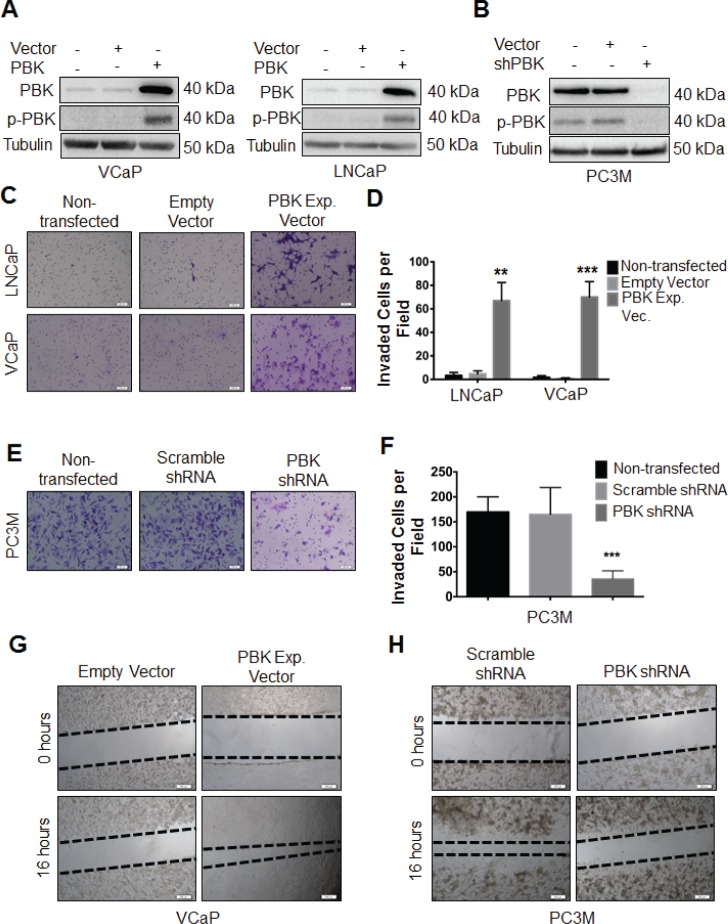
PBK causally modulates invasive and migratory potential of prostate cancer cells (**A**) Ectopic expression of PBK in hormone-sensitive LNCaP and VCaP cells is measured by Western blot analysis. Non-transfected and empty vector-infected cells were used as controls. (**B**) Western blot showing knockdown of PBK in PC-3M cells. Non-transfected and scrambled shRNA-transfected cells were used as controls. Representative images of (**C**) LNCaP or VCaP cells, either appropriate controls or stably overexpressing PBK, stained with crystal violet after being subjected to a modified Boyden chamber invasion assay, in addition to (**E**) PC-3M cells, with PBK expression stably knocked down and appropriate controls. (**D** and **F**) Quantification of cells that had invaded from three different experiments. Invaded cells were counted in four fields of view from each experiment. Quantitative data are represented as SEM ± SE. ** represents a *p*-value <0.01 and *** a *p*-value <0.001. Representative images of cell migration 16h after creation of a wound in (**G**) VCaPPBK and (H) PC-3M-shPBK stably modified cells compared to their respective controls (empty vector or scrambled-shRNA stable cells, respectively). The black dotted line indicates the boundary of the area covered by cells. *n* ≥ 3.

