# Supplementary material for: PBK/TOPK enhances aggressive phenotype in prostate cancer via β-catenin-TCF/LEF-mediated matrix metalloproteinases production and invasion
Source: Oncotarget. 2015 Mar 30;6(17):15594–609. doi: 10.18632/oncotarget.3709 (PMC4558173; doi:10.18632/oncotarget.3709)
Supplement: Supplementary file 1 [file oncotarget-06-15594-s001.pdf]

PBK/TOPK enhances aggressive phenotype in prostate cancer via β-catenin-TCF/LEF-mediated matrix metalloproteinases production and invasion

Supplementary Material

Supplementary Materials and Methods

PCR Primers

	Sequence	Conditions
Quantitative PCR primers		Tm 56° C
MMP2-Fw	TACAGGATCATTGGCTACACACC	
MMP2-Rev	GGTCACATCGCTCCAGACT	
MMP9-Fw	TGTACCGCTATGGTTACACTCG	
MMP9-Rev	GGCAGGGACAGTTGCTTCT	
GAPDH-Fw	GAAGGTGAAGGTCGGAGTC	
GAPDH-Rev	GAAGATGGTGATGGGATTTC	
Semi-quantitative PCR primers		
PBK-Fw	GCCAATGATGGCAGTCTGTG	Tm 56° C, 30 cycles
PBK-Rev	GTGCAGATACTTTAACCCTCTTGC	

MTT Assay

Equal numbers of cells were plated in 96-well plates. Stably genetically modified cells were used or cells were treated with the indicated doses of HI-TOPK-032 for 72 hours. At the end of the treatment period, 10 µl of MTT reagent (Life Technologies) was added to each well and cells were incubated at 37°C for 4 hrs. Isopropanol:HCl solution (100:1) was added to each well (100 µl) and mixed thoroughly, following which absorbance was measured at 570nm with a reference filter of 655nm.

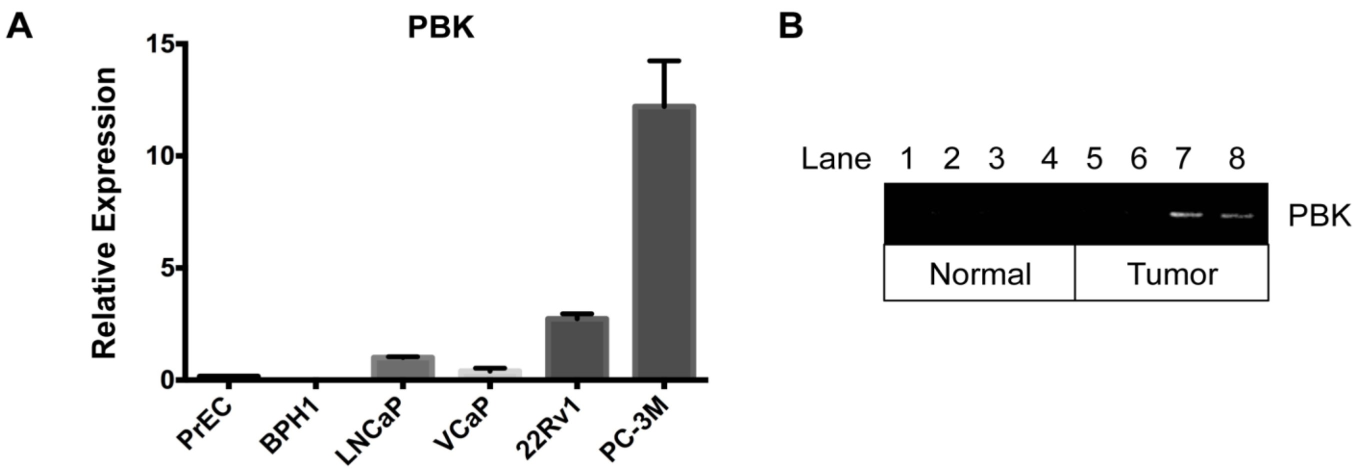

Supplementary Figure 1. PBK gene expresion in human prostate cell lines and clinical samples. (A) Quantitative PCR analysis of PBK expression in a panel of prostate cell lines. (B) One microgram of RNA from patient non-tumor (lanes 1-4) and tumor samples (lanes 5-8) was subjected to a one-step, semi-quantitative RT-PCR to analyze relative PBK expression.

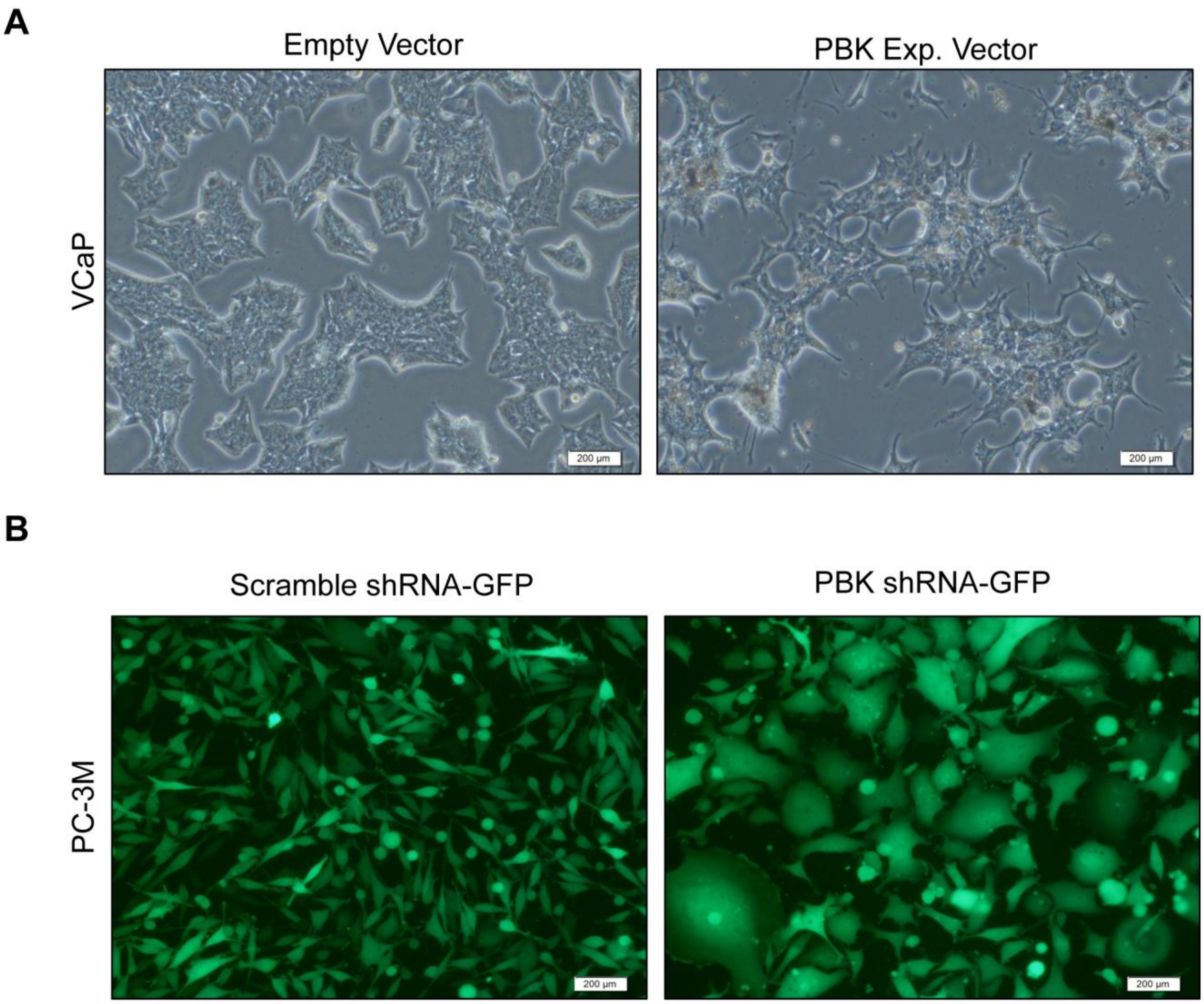

Supplementary Figure 2. Overexpression or genetic knockdown of PBK in human prostate cancer cells resulted in morphological changes. (A) VCaP cells stably transfected with empty vector or PBK expression vector. VCaP PBK-overexpressing cells show more projections at the periphery of the colony compared to VCaP empty vector cells. (B) PC-3M cells stably expressing a scramble shRNA-GFP or PBK-shRNA-GFP construct were visualized using fluorescent microscopy. PBK knockdown PC-3M cells are more flat and broad compared to scrambled shRNA-GFP transfected cells. Scale bars are 200 µm.

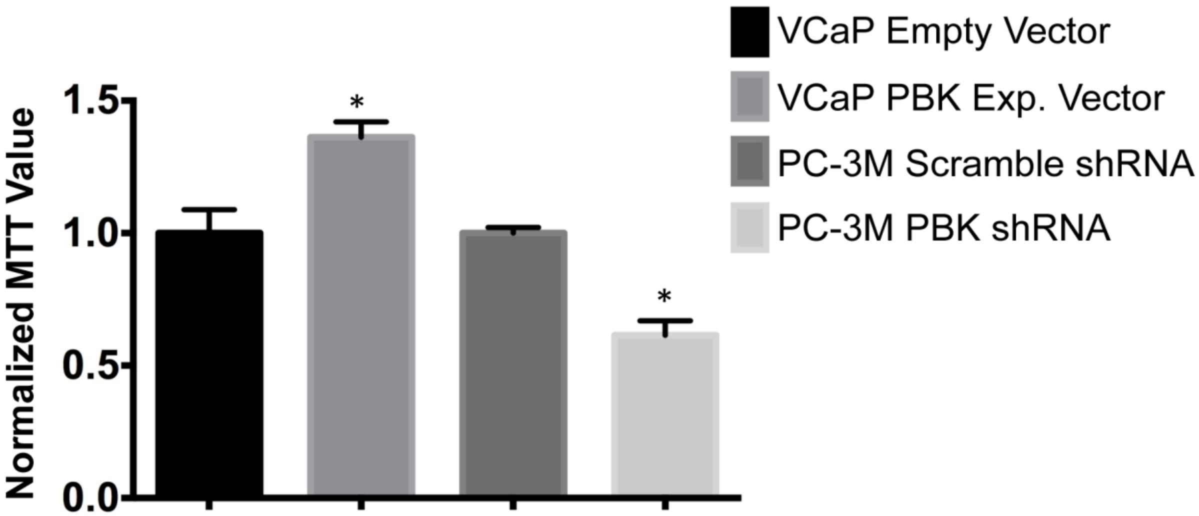

Supplementary Figure 3. PBK modulates growth of prostate cancer cell lines. MTT assays were performed 72 hours after plating stably genetically modified VCaP and PC-3M cells. Normalized MTT values showing that PBK overexpression in VCaP cells significantly increases growth compared to empty vector stable cells, whereas, PBK knockdown slows growth in PC-3M cells compared to scrambled shRNA transfected cells . Data are mean ± SE. * indicates a p-value <0.05.

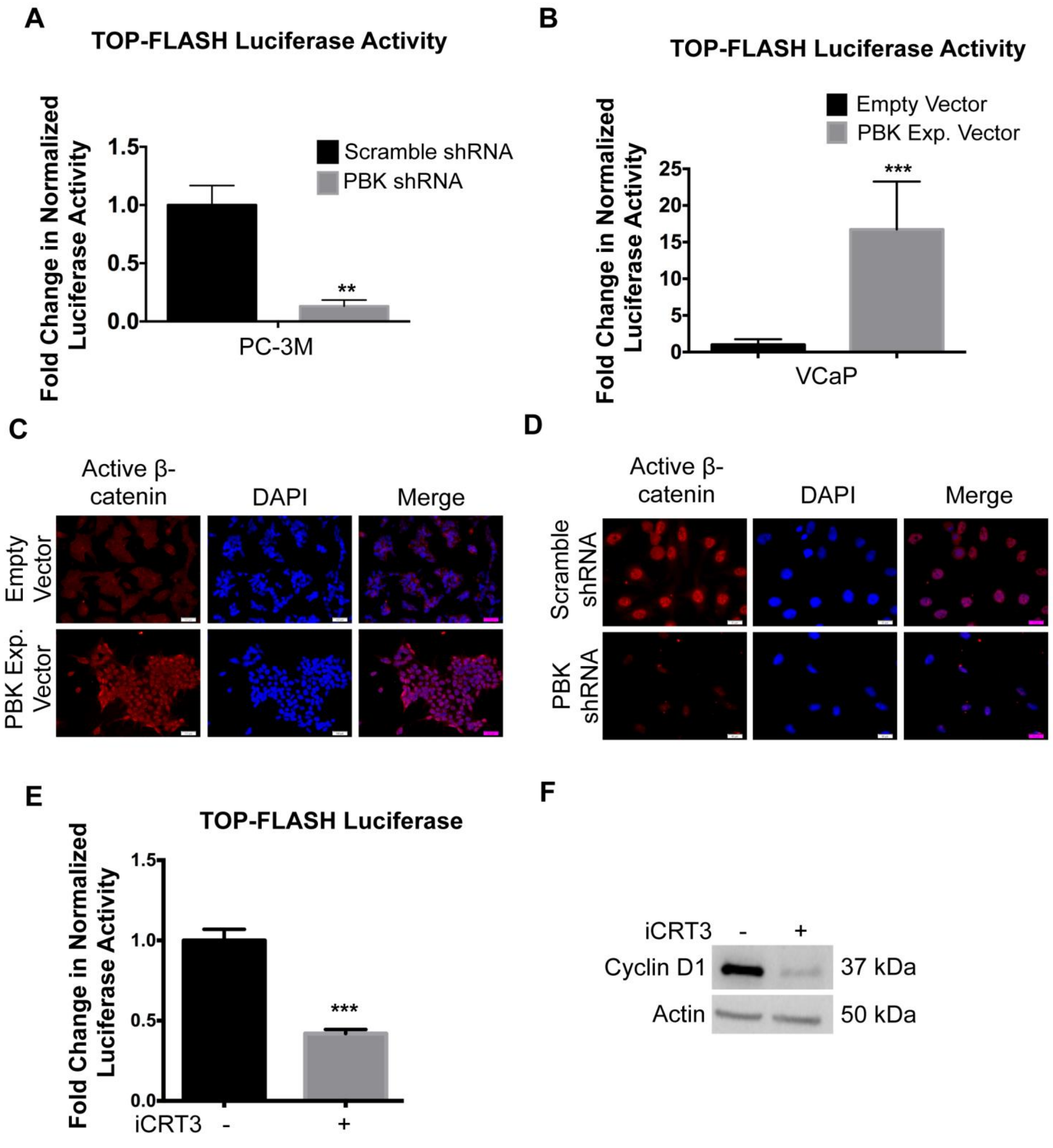

Supplementary Figure 4. PBK regulates β-catenin-TCF/LEF signaling. Transcriptional activity of TCF/LEF transcription factors was measured by transient transfection of stably modified **(A)** PC-3M and **(B)** VCaP cells with the TOP-FLASH luciferase reporter construct. Immunofluorescence of stably modified (**C**) VCaP and (**D**) PC-3M cells showing localization of active (non-phosphorylated) β-catenin. Scale bars are 100 µm. **(E)** VCaP cells stably expressing TOP-FLASH were treated with 50 µM iCRT3 to show inhibition of the Wnt signaling pathway. **(F)** Western blot analyses showing that treatment of VCaP cells with iCRT3 decreases protein levels of a Wnt target gene. Data are mean ± SE. **; p-value < 0.01; ***; p-value < 0.001.

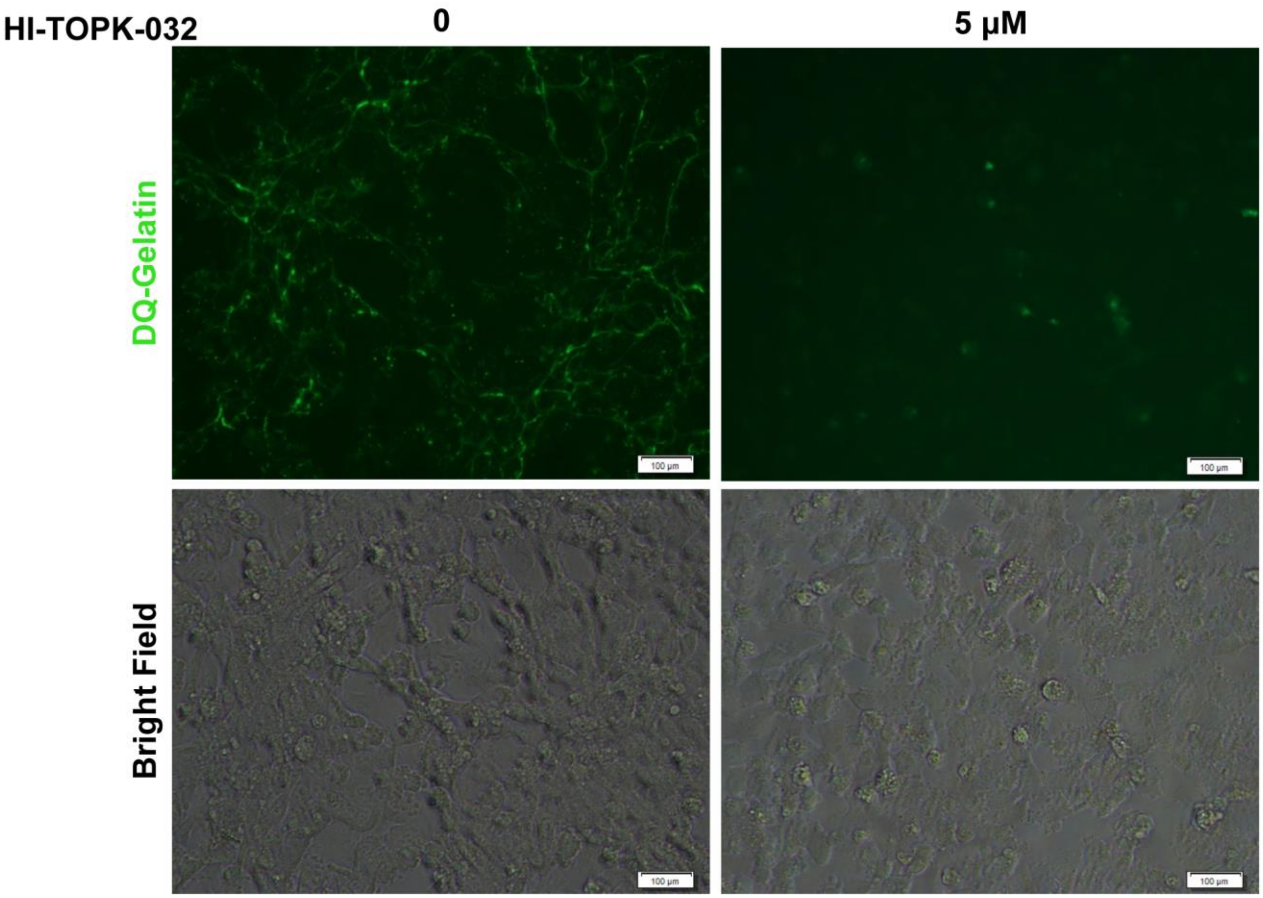

Supplementary Figure 5. HI-TOPK-032 treatment reduces MMP-2 and MMP-9 production. In situ DQ gelatin staining of HT1080 cells treated with vehicle or 5 µM HI-TOPK-032. HT1080 cells were pre-treated with DMSO (control) or HI-TOPK-032 for 24 hours, then DQ gelatin was added to HT1080 cells in culture for an hour. MMPs enzymatically cleave DQ-gelatin, yielding highly fluorescent fragments. Representative pictures were taken under Olympus fluorescent microscope (upper panels) and bright field images (lower panels) at same magnification.

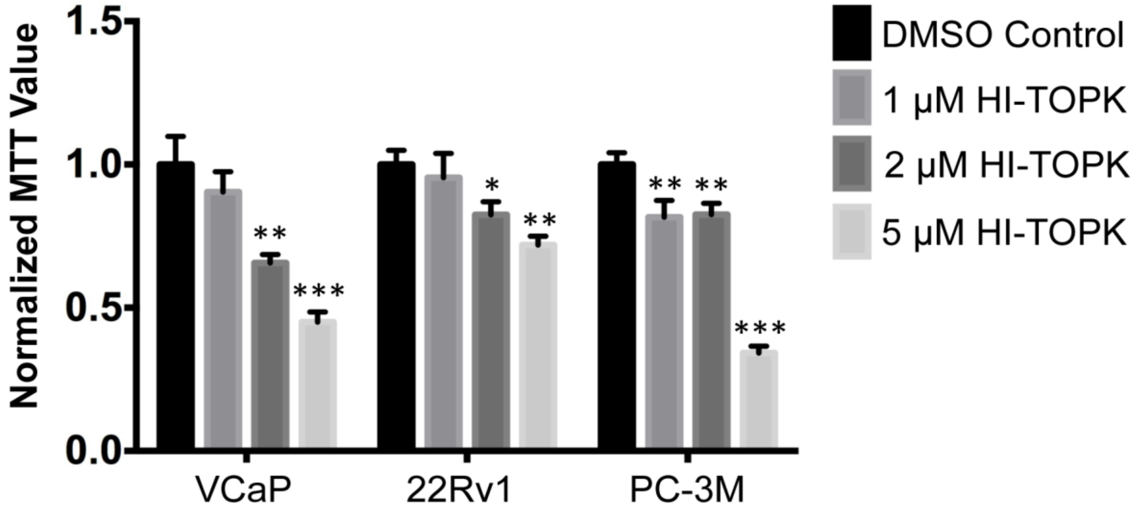

Supplementary Figure 6. Pharmacological inhibition of PBK with HI-TOPK-032 suppressed growth of various prostate cancer cell lines. MTT assays were performed using various concentrations of HI-TOPK-032, with DMSO as control, in three different prostate cancer cell lines. Normalized MTT value showing growth inhibition of VCaP, 22Rv1 and PC-3M cells after treatment with HI-TOPK-032 for 72 hours. Data are mean ± SE. * indicates a p-value <0.05; ** p-value <0.01; *** p-value <0.001.

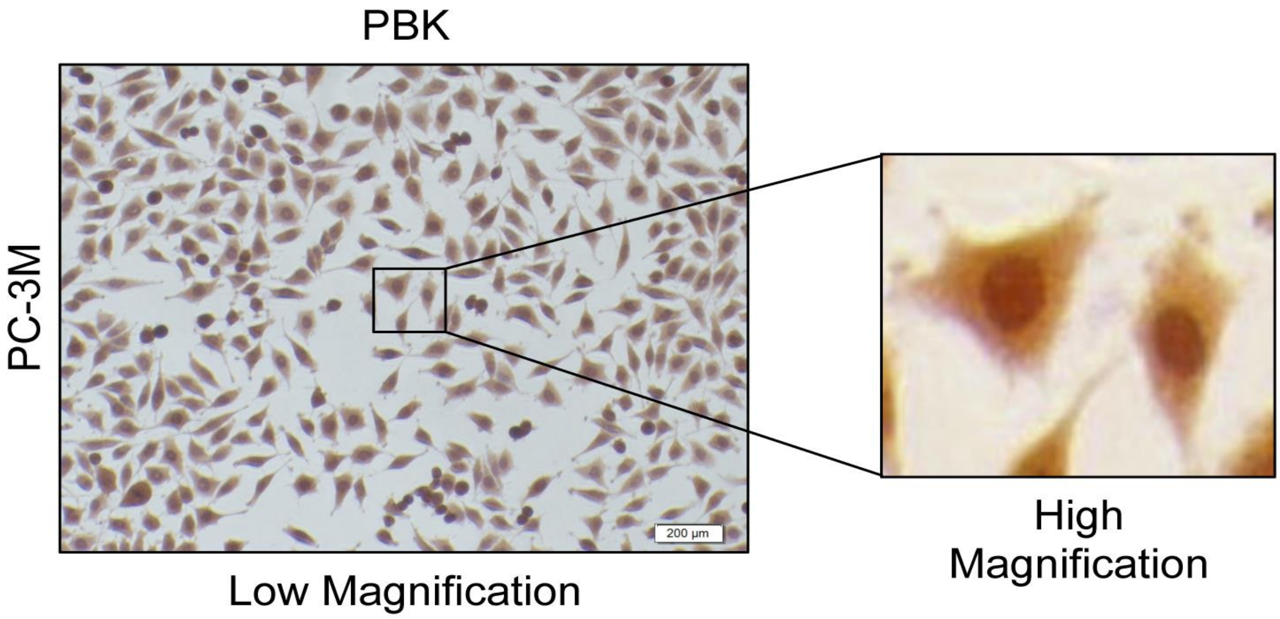

Supplementary figure 7. PBK localization in PC-3M cells bymmunocytochemistry. PC-3M cells were plated on ECL-coated chambered slides and 24 hours after plating, cells were fixed in chilled methanol at -20^{0}C for 30 min. Fixed cells were air dried and then rehydrated with PBS, blocked with 0.1% BSA and incubated with PBK antibody for 24h at 4^{0}C. After being washed, cells were incubated with secondary antibody, ABC complex and stained with DAB substrate. PBK protein was localized predominantly in the nucleus but substantial amounts of PBK were also present in cytoplasm. A representative picture shown at lower and higher magnifications.
